# A combined electrophysiological and morphological study of neuropeptide Y–expressing inhibitory interneurons in the spinal dorsal horn of the mouse

**DOI:** 10.1097/j.pain.0000000000000407

**Published:** 2015-11-04

**Authors:** Noboru Iwagaki, Robert P. Ganley, Allen C. Dickie, Erika Polgár, David I. Hughes, Patricia Del Rio, Yulia Revina, Masahiko Watanabe, Andrew J. Todd, John S. Riddell

**Affiliations:** a Spinal Cord Group, Institute of Neuroscience and Psychology, College of Medical, Veterinary and Life Sciences, University of Glasgow, Glasgow, United Kingdom; b Department of Anatomy, Hokkaido University School of Medicine, Sapporo, Japan

**Keywords:** Spinal cord, Whole-cell recording, Green fluorescent protein, Confocal microscopy, Capsaicin

## Abstract

The spinal dorsal horn contains numerous inhibitory interneurons that control transmission of somatosensory information. Although these cells have important roles in modulating pain, we still have limited information about how they are incorporated into neuronal circuits, and this is partly due to difficulty in assigning them to functional populations. Around 15% of inhibitory interneurons in laminae I-III express neuropeptide Y (NPY), but little is known about this population. We therefore used a combined electrophysiological/morphological approach to investigate these cells in mice that express green fluorescent protein (GFP) under control of the NPY promoter. We show that GFP is largely restricted to NPY-immunoreactive cells, although it is only expressed by a third of those in lamina I-II. Reconstructions of recorded neurons revealed that they were morphologically heterogeneous, but never islet cells. Many NPY-GFP cells (including cells in lamina III) appeared to be innervated by C fibres that lack transient receptor potential vanilloid-1, and consistent with this, we found that some lamina III NPY-immunoreactive cells were activated by mechanical noxious stimuli. Projection neurons in lamina III are densely innervated by NPY-containing axons. Our results suggest that this input originates from a small subset of NPY-expressing interneurons, with the projection cells representing only a minority of their output. Taken together with results of previous studies, our findings indicate that somatodendritic morphology is of limited value in classifying functional populations among inhibitory interneurons in the dorsal horn. Because many NPY-expressing cells respond to noxious stimuli, these are likely to have a role in attenuating pain and limiting its spread.

## Abstract 2

Neuropeptide Y–expressing spinal inhibitory interneurons are morphologically diverse and include cells innervated by transient receptor potential vanilloid-1–negative C fibres and a subset that targets lamina III projection neurons.

## 1. Introduction

Inhibitory interneurons, which account for ∼30% of the neurons in laminae I-III of the spinal dorsal horn,^44^ are important in controlling the transmission of sensory inputs perceived as pain or itch^2,11,12,31,51,61^ and represent potential targets for treatments designed to relieve these symptoms. Despite intensive investigation, we still know relatively little about their organisation or about the synaptic circuits through which they modulate sensory transmission. This is largely due to the difficulty in identifying distinct populations among these cells.^14,61^ A widely used morphological classification scheme for lamina II interneurons defines 4 major classes: islet, central, vertical, and radial,^15^ with islet cells and some central cells corresponding to inhibitory interneurons.^76^ However, most studies of lamina II neurons have found that ∼25% of these cells could not be classified morphologically,^15,19,76^ and little is known about the relation between morphology and function for interneurons in laminae I and III.^49,54^

The complex neurochemistry of the dorsal horn provides an alternative way of defining neuronal populations, and we have identified 4 largely nonoverlapping classes among the inhibitory interneurons in laminae I-III, defined by the expression of neuronal nitric oxide synthase (nNOS), galanin, neuropeptide Y (NPY), and parvalbumin.^46^ These classes differ in their responses to noxious stimuli and are believed to have different roles in modulating sensory transmission. For example, parvalbumin cells are involved in presynaptic inhibition of low-threshold mechanoreceptive afferents,^24^ whereas the galanin cells, which also express dynorphin,^3,52^ may contribute to prevention of mechanical allodynia.^11^ In addition, it has been suggested that the nNOS and/or galanin populations play a part in inhibiting itch.^31^

Little is known about the NPY-expressing cells, although NPY itself has a complex role in nociception.^4^ For example, NPY acting at the spinal level has been reported to increase thermal nociceptive thresholds in naive animals^21^ and to reduce hyperalgesia in both inflammatory and neuropathic models.^26,60^ However, it has also been found that NPY can exacerbate hyperalgesia after peripheral nerve injury.^65,70^ The NPY-expressing cells are all GABA immunoreactive and account for ∼15% of the inhibitory interneurons in laminae I-III in the rat.^47^ It has been shown in the mouse that their development is dependent on the transcription factors Ptf1a and Pax2, which determine inhibitory fate,^3,22,71^ and that they represent a developmentally distinct population among the inhibitory interneurons.^3^ We have previously shown that many NPY^+^ neurons in the rat respond to noxious stimuli^46^ and that NPY-immunoreactive axons, presumably derived from local NPY-expressing interneurons, selectively innervate a population of anterolateral tract (ALT) projection neurons in lamina III.^5,47,48^ In this study, we have investigated NPY-expressing interneurons in laminae I-III of the mouse. Specifically, we tested whether these cells correspond to any of the morphologically defined classes^15^ and whether they are innervated by unmyelinated primary afferents, most of which are nociceptors. We also looked for evidence that the NPY cells that innervate lamina III ALT neurons represent a distinct subpopulation.^47^

## 2. Methods

Experiments were approved by the Ethical Review Process Applications Panel of the University of Glasgow and were performed in accordance with the U.K. Animals (Scientific Procedures) Act 1986.

The mouse line B6.FvP-Tg(Npy-hrGFP)1Lowl/J (The Jackson Laboratory, Stock number 006417),^67^ which expresses humanized Renilla green fluorescent protein (GFP) under control of the NPY promoter, was used for most of the experiments in this study. These mice (which will be referred to as NPY-GFP) were maintained as heterozygous (University of Glasgow Biological Services) by crossing with wild-type C57Bl/6 mice, and the resulting offspring were genotyped using transcranial illumination to visualise GFP expression in the brain at P3-4.

### 2.1. Immunocytochemical assessment of NPY-GFP mice

Five NPY-GFP mice of either sex, aged 27 to 42 days (13-17 g, similar to the age range used in the electrophysiological studies), were deeply anaesthetised with pentobarbitone (30 mg intraperitoneally) and perfused through the left ventricle with a fixative that contained 4% freshly depolymerised formaldehyde. Spinal cord segments L3-L4 were postfixed overnight and cut into transverse sections 60 μm thick with a vibrating blade microtome VT1200 or VT1000S, Leica, Milton Keynes, United Kingdom). The sections were then processed for immunocytochemistry, as described previously.^13^ In all cases, sections were incubated in primary antibodies for 3 days and in secondary antibodies for 1 day, in both cases at 4°C. All antibodies were diluted in phosphate-buffered saline (PBS) that contained 0.3 M NaCl, 0.3% Triton-X100, and 5% normal donkey serum. Species-specific secondary antibodies (Jackson ImmunoResearch, West Grove, PA) were raised in donkey and conjugated to Rhodamine Red, DyLight 649, Alexa 647, Pacific Blue, or biotin. Biotinylated secondary antibodies were revealed by incubation for 2 to 4 hours in avidin conjugated to Pacific Blue (Life Technologies, Paisley, United Kingdom).

Sections were reacted with each of the following combinations of primary antibodies: (1) rabbit anti-NPY and mouse monoclonal antibody NeuN; (2) rabbit anti-galanin, sheep anti-nNOS, and guinea-pig anti-parvalbumin. Sections from 3 mice were reacted for each antibody combination, and those from the first combination were counterstained with 4′,6-diamidino-2-phenylindole to reveal nuclei. The sources of the primary antibodies and their dilutions are shown Table 1. After completion of immunoreactions, sections were mounted in antifade medium and stored at −20°C.

**Table 1:**
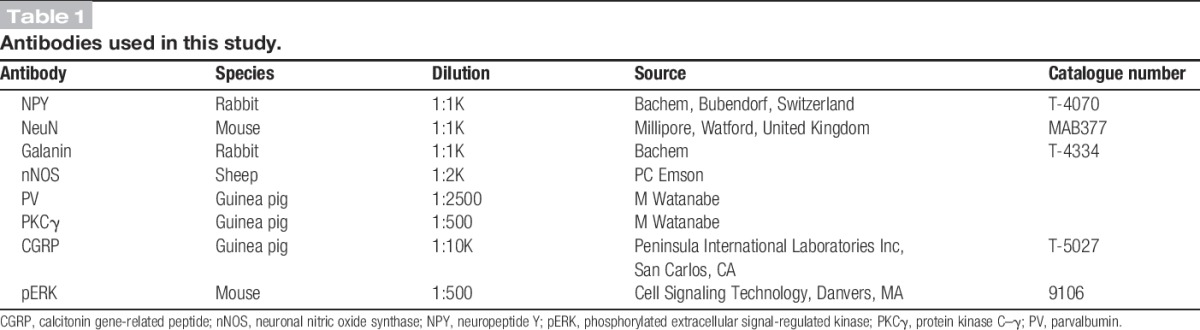
Antibodies used in this study.

Sections were scanned with a Zeiss LSM 710 confocal microscope equipped with argon multiline, 405 nm diode, 561 nm solid state, and 633 nm HeNe lasers, and a spectral detection system. In each case, confocal image stacks (z-separation of 1 or 2 μm) were obtained through a 40× oil-immersion lens (numerical aperture 1.3) with the aperture set to 1 Airy unit. The resulting z-stacks were analysed with Neurolucida for Confocal software (MBF Bioscience, Williston, VT).

To assess the extent of overlap between GFP and NPY, confocal z-stacks obtained from a single section (scanned at 1 μm z-separation) from each of 3 mice were analysed using a modification of the optical disector method.^45^ In each series, the 14th optical section was designated as the reference section and the 35th as the look-up. Each optical section in the z-series was examined, and the locations of all neuronal nuclei that were NPY immunoreactive and/or GFP^+^ and were present in the reference section or appeared in subsequent sections in the series were plotted onto an outline of the dorsal horn. All of those that were still present on the look-up section were then excluded, leaving only those neurons for which the bottom surface of the nucleus was located between reference and look-up sections.

To determine whether any of the GFP^+^ neurons were immunoreactive for galanin, nNOS, or parvalbumin, 2 sections from each of 3 mice were scanned through their full thickness (at 2 μm z-separation) and the channel corresponding to GFP was initially viewed. All GFP^+^ neurons in laminae I-III were plotted onto an outline drawing. The other channels were then viewed, and the presence or absence of galanin, nNOS, or parvalbumin immunoreactivities was noted.

### 2.2. Slice preparation and electrophysiology

Spinal cord slices were obtained from 68 NPY-GFP mice 4 to 6 weeks old of either sex. As described previously,^10,13,27^ the lumbar spinal cord was isolated either after laminectomy performed during anaesthesia with isoflurane (1%-3%) or else in ice-cold dissection solution after decapitation under brief isoflurane anaesthesia. Mice from which the spinal cord had been removed under anaesthesia were then immediately decapitated. The spinal cord was transferred to ice-cold dissecting solution containing the following (in mM): 3.0 KCl, 1.2 NaH_2_PO_4_, 0.5 to 2.4 CaCl_2_, 1.3 to 7.0 MgCl_2_, 26.0 NaHCO_3_, 15.0 to 25.0 glucose, 240.0 to 251.6 sucrose, oxygenated with 95% O_2_ and 5% CO_2_, and the dura and pia mater were removed. For experiments that involved dorsal root stimulation, the L4 and L5 dorsal roots (with ganglia removed) were left attached to the cord. The spinal cord was cut into parasagittal (300-500 μm) or transverse (350 μm) slices with a vibrating blade microtome (MicromHM 650V; Fisher Scientific, Loughborough, United Kingdom), and these were then allowed to recover at room temperature for at least 30 minutes in recording solution that contained the following (in mM): 125.8 to 127.0 NaCl, 3.0 KCl, 1.2 NaH_2_PO_4_, 2.4 CaCl_2_, 1.3 MgCl_2_, 26.0 NaHCO_3_, 15 glucose, oxygenated with 95% O_2_ and 5% CO_2_. Although most recordings were made in parasagittal slices, for some experiments involving dorsal root stimulation, transverse sections were used as this method of cutting can generate more slices with attached dorsal roots.

Targeted whole-cell patch-clamp recordings were made from GFP-positive neurons that were visualized under fluorescence and infrared differential interference contrast microscopy on an Olympus BX51WI microscope. Patch pipettes were pulled with a horizontal puller (P-97; Sutter Instruments, Novato, CA) from glass capillaries (World Precision Instruments or Harvard Apparatus). Typical pipette electrical resistance was 4 to 7 MΩ when filled with internal solution, which usually contained the following (in mM): 130.0 potassium gluconate, 10.0 KCl, 2.0 MgCl_2_, 10.0 HEPES, 0.5 EGTA, 2.0 ATP-Na_2_, 0.5 GTP-Na, and pH adjusted to 7.3 with 1.0 M KOH. In some experiments that involved dorsal root stimulation, internal solution containing the following (in mM): 120.0 Cs-methylsulfonate, 10.0 Na-methylsulfonate, 10.0 EGTA, 1.0 CaCl_2_, 10.0 HEPES, 5.0 QX-314-Cl[2(triethylamino)-N-(2,6-dimethylphenyl) acetamine chloride], 2.0 Mg_2_-ATP, pH adjusted to 7.2 with CsOH was used instead. In all cases, Neurobiotin (0.2%; Vector Laboratories, Peterborough, United Kingdom) was included in the internal solution for subsequent morphological analysis of recorded cells. Patch-clamp signals were amplified and filtered (4 kHz low-pass Bessel filter) with a MultiClamp 700B amplifier (Molecular Devices) and acquired at 10 kHz using a Digidata 1440A or 1550 A/D board and pClamp 10 software (Molecular Devices, Wokingham, United Kingdom).

After successful configuration of whole-cell mode, cells were voltage clamped at −60 mV. Short voltage pulses (100 milliseconds, −70 to −50 mV, 2.5 mV increments) were delivered to generate current–voltage (*I*-*V*) relationships for recorded cells, and those that exhibited resting membrane potentials more depolarised than −30 mV were not analysed further. Current-clamp mode was used to examine the pattern of action potential firing. Cells were sometimes presented with continuous bias currents to return membrane potentials to around −60 mV, and depolarising square current pulses (1 second) of increasing amplitude were applied.

To investigate primary afferent input to the neurons, evoked excitatory postsynaptic currents (eEPSCs) were recorded in spinal cord slices with attached dorsal roots in response to dorsal root stimulation, as described previously.^13,63,64^ Cells were voltage clamped at −70 mV, and the dorsal root was stimulated with a suction electrode. To determine the fibre types providing input to the recorded neurons, stimuli were initially applied at low frequency (0.05 Hz, stimulus duration 0.1 milliseconds, ×3) using an ISO-Flex stimulus isolator (AMPI Intracell), with progressively increasing intensities. The stimulation intensities used were 25 μΑ for Aβ fibres, 100 μA for Aδ fibres, and 500μA and 1 mA for C fibres. Cells in which no monosynaptic response was evident at 1 mA were additionally stimulated at 3 and/or 5 mA. Primary afferent input was characterized as monosynaptic or polysynaptic in the manner of Nakatsuka et al,^41^ as described previously.^63,64^ Dorsal roots were stimulated 20 times at 20 Hz for Aβ fibres, 2 Hz for Aδ fibres, and 1 Hz for C fibres. A-fibre responses were considered monosynaptic if there was an absence of failures and a latency variability of ≤2 milliseconds, whereas C-fibre responses were classified as monosynaptic if there was an absence of failures, regardless of whether the latency was variable. The estimated conduction velocity for monosynaptic primary afferent inputs was calculated on the basis of the response latency, measured as the time between the stimulus artefact and the onset of the monosynaptic eEPSC, and the length of the stimulated dorsal root, measured as the distance between the stimulation electrode and the dorsal root entry zone.

To provide information about monosynaptic inputs from primary afferents that express transient receptor potential channels and to allow a comparison with a different population of inhibitory interneurons (those that express GFP under control of the prion promoter in the PrP-GFP mouse line; PrP-GFP cells)^13^ miniature excitatory postsynaptic currents (mEPSCs) were recorded in the presence of tetrodotoxin (0.5 μM), bicuculline (10 μM), and strychnine (5 μM). While the cell was voltage clamped at −60 mV, capsaicin (2 μM) or icilin (20 μM) was bath applied through 3-way stopcocks without any change in perfusion rate (approximately 2 mL/min). For experiments with icilin, bath temperature was raised to 32°C with an in-line heating perfusion tube (HPT-2; ALA Scientific Instruments, Farmingdale, NY) and a control system (PCT-10, NPI), as described previously.^13^ Voltage-clamp recordings were obtained throughout the period before and during drug application, and mEPSCs were detected off-line using Mini Analysis Program software (Synaptosoft, Decatur, GA). The period for analysis was set to 5 minutes for capsaicin application and 3 minutes for icilin application, and in both cases, the period of analysis began 2 to 3 minutes after the stopcock was opened, to allow the drugs to reach the recording chamber (which takes ∼2 minutes).

For a subset of cells that received monosynaptic C-fibre input, the capsaicin sensitivity of this input was assessed.^66,74^ Monosynaptic C-fibre eEPSCs were evoked at 1 mA (0.05 Hz, 0.1 milliseconds stimulus duration) for 10 minutes (baseline) followed by a further 10 minutes in the presence of capsaicin (2 μM). To determine whether an individual cell received monosynaptic C-fibre input that was capsaicin sensitive, peak monosynaptic C-fibre eEPSC amplitude was measured for each sweep, and the amplitude of sweeps recorded in the 3 minutes before capsaicin application was compared with those during the final 3 minutes of capsaicin application. Tetrodotoxin and icilin were obtained from Tocris Bioscience, and 1(S),9(R)-(−)-bicuculline methiodide, strychnine hydrochloride, and capsaicin were from Sigma-Aldrich.

After electrophysiological recording, slices were immersion fixed overnight in 4% formaldehyde at 4°C.

### 2.3. Morphological analysis of recorded neurons

The processing of slices that contained recorded cells and the reconstruction of these cells with Neurolucida for Confocal software (MBF Bioscience) were similar to that described previously.^13^ After fixation, slices containing recorded cells were rinsed in PBS and incubated overnight at 4°C in avidin Rhodamine (1:1000; Jackson ImmunoResearch) diluted in PBS containing 0.3% Triton X-100. They were then mounted on slides and scanned with the confocal microscope. Confocal image stacks of filled cells were acquired by scanning through a 63× oil-immersion lens (numerical aperture 1.4) with 0.5 µm z-spacing and the aperture set to 1 Airy unit. Initial scans included all dendritic trees and axonal arbors that were visible at this stage, and these were analysed offline. In all cases, the presence of GFP was confirmed by scanning for the native protein within the cell bodies of the filled neurons. Axons could readily be distinguished from dendrites because they were generally thinner, showed little tapering at increasing distance from the soma, lacked spines, and possessed numerous irregularly spaced varicosities.^13,15,76^

Initially, the dendritic trees and axonal arbors of the cells were manually reconstructed with the neuron tracing feature of Neurolucida. Slices were then flat embedded in agar and resectioned at 60 μm with a vibrating blade microtome (Leica VT1200), and the sections were kept in serial order. Sections that contained parts of the dendritic or axonal tree that were deep within the slice and had not previously been visible were scanned, and these were added to the reconstruction. To determine laminar boundaries, 1 section from each slice was immunostained to reveal protein kinase C–γ (PKCγ), which is present in a plexus of dendrites that occupies the inner half of lamina II (IIi).^23^ The boundaries between the outer part of lamina II (IIo) and lamina IIi and between laminae IIi and III were added to the reconstructions, by aligning sections containing the recorded cells with nearby sections stained for PKCγ. The lamina I/IIo border was taken to be 20 μm below the dorsal white matter,^13^ and this was also added to the reconstruction. Morphometric data for cell bodies, dendritic trees, and axonal arbors of the reconstructed cells were obtained from Neurolucida Explorer.

To allow a comparison of the somatodendritic morphology of NPY-GFP cells with that of a neurochemically distinct group of inhibitory interneurons, we also analysed morphometric data obtained from PrP-GFP cells.^13,17,18^ In the PrP-GFP mouse, GFP is found exclusively in inhibitory interneurons in the dorsal horn and is virtually restricted to those that express nNOS and/or galanin.^27^ We performed a cluster analysis with Ward's method,^69^ using 55 morphological parameters (6 for the soma and 49 for the dendritic tree) that were obtained from the Neurolucida reconstructions. Because the PrP-GFP cells are mainly present in lamina II, this analysis was performed on those NPY-GFP cells with well-labelled dendritic trees and cell bodies in lamina II (n = 20), together with a population of 70 lamina II PrP-GFP cells that were recorded during a previous study from our laboratory.^13^ To reduce the dimensionality of the original data set while preserving variance, principal components were calculated from the data set with the factor analysis function of SPSS software (IBM). The number of principal components to be retained for cluster analysis was then determined from a scree test.

To determine whether the recorded neurons included those that innervate lamina III ALT neurons,^47,48^ we selected 38 of the cells and identified sections that contained a significant part of their axonal arbor. These sections were immunostained with rabbit anti-NPY and guinea pig antibody against calcitonin gene-related peptide (CGRP), which were revealed with Alexa 647 and Pacific Blue, as described above. This combination of antibodies was chosen because both of these peptides are present in bundles of axons that are associated with the cell bodies and dendrites of the lamina III ALT neurons.^5^ We also tested for the presence of detectable NPY immunoreactivity in axons of the filled cells. These were defined as NPY-immunoreactive if there was immunostaining for the peptide in at least 5 boutons.^13^

### 2.4. pERK in NPY-immunoreactive neurons

To test whether any NPY-expressing lamina III neurons in the mouse respond to noxious mechanical stimuli, we performed immunocytochemistry for phosphorylated extracellular signal-regulated kinases (pERK), a well-established marker of neuronal activation,^28,29^ on transverse sections of spinal cord from 3 male wild-type C57Bl/6 mice (17 g) that had received a pinch stimulus to the left hindpaw as part of a previous study.^57^ These animals were initially anaesthetised with isoflurane and maintained with 10% urethane (intraperitoneally). Folds of skin on the plantar surface of the hindpaw (over the tarsal bones) were pinched at 10 locations (5 seconds each) with watchmakers' forceps, and 5 minutes after the last stimulus the animals were perfused with 4% formaldehyde. Sections from the lumbar enlargement were reacted with antibodies against pERK, NPY, and PKCγ, which were revealed with fluorescent secondary antibodies. Three or 4 sections that contained numerous pERK^+^ cells were selected from each animal (before NPY immunostaining was viewed) and scanned with the confocal microscope through the 40× lens to produce z-stacks (2 μm z-separation) through the full thickness of the tissue. The z-scans were analysed with Neurolucida, and the band of PKCγ-immunoreactive dendrites was used to define the laminae II-III border, while the depth of lamina III was taken to be 100 μm. We used phosphorylation of ERK, rather than the transcription factor Fos,^25^ to identify activated cells because we have found that this method results in more consistent labelling in the dorsal horn after noxious mechanical stimuli.^43,46^

### 2.5. Antibody characterisation

The NPY antibody was raised against synthetic NPY, and we have previously reported that staining is abolished by pretreatment with NPY.^50^ In addition, we have found that staining is absent in mice in which NPY has been knocked out^56^ (AJT and H Herzog, unpublished observations). The NeuN antibody was raised against cell nuclei extracted from mouse brain and found to react with a protein specific for neurons,^38^ which has subsequently been identified as the splicing factor Fox-3.^32^ The galanin antibody was raised against the synthetic peptide, and staining is absent in the brains of galanin-knockout mice.^35^ The nNOS antibody was raised against purified recombinant rat nNOS and labels a band of 155 kDa in Western blots of rat hypothalamus, while immunostaining is abolished by preabsorption with nNOS.^20^ The parvalbumin antibody is directed against the mouse protein and recognises a band of 13 kDa on Western blots of mouse brain homogenates.^40^ The PKCγ antibody, which was raised against amino acids 648 to 697 of the mouse protein, detects a single band at 75 kDa in wild-type (but not PKCγ^−/−^) mice and stains identical structures to those detected by a well-characterised rabbit antibody.^53,77^ The CGRP antibody detects both α and β forms of the peptide (manufacturer's specification). The monoclonal pERK antibody detects both ERK1 and ERK2 that are dually phosphorylated at Thr202 and Tyr204 sites and does not cross-react with either JNK or p38 MAP kinase that is phosphorylated at the corresponding sites (manufacturer's specification). Staining with this antibody was restricted to somatotopically appropriate areas of the dorsal horn after noxious stimulation.

### 2.6. Statistics

Miniature EPSC interevent intervals in control conditions were compared to those in the presence of drugs by the Kolmogorov–Smirnov 2-sample test. Dorsal root eEPSC amplitudes in the presence or absence of capsaicin were compared with the Wilcoxon matched-pairs signed-rank test. Dendritic spine density for lamina II and lamina III neurons was compared with the Mann–Whitney U test, while other differences in anatomical properties of neurons in the 2 laminae were compared with unpaired t-tests. In all cases, a *P* value <0.05 was considered significant.

## 3. Results

### 3.1. Expression of GFP in the NPY-GFP mouse dorsal horn

**Figure 1:**
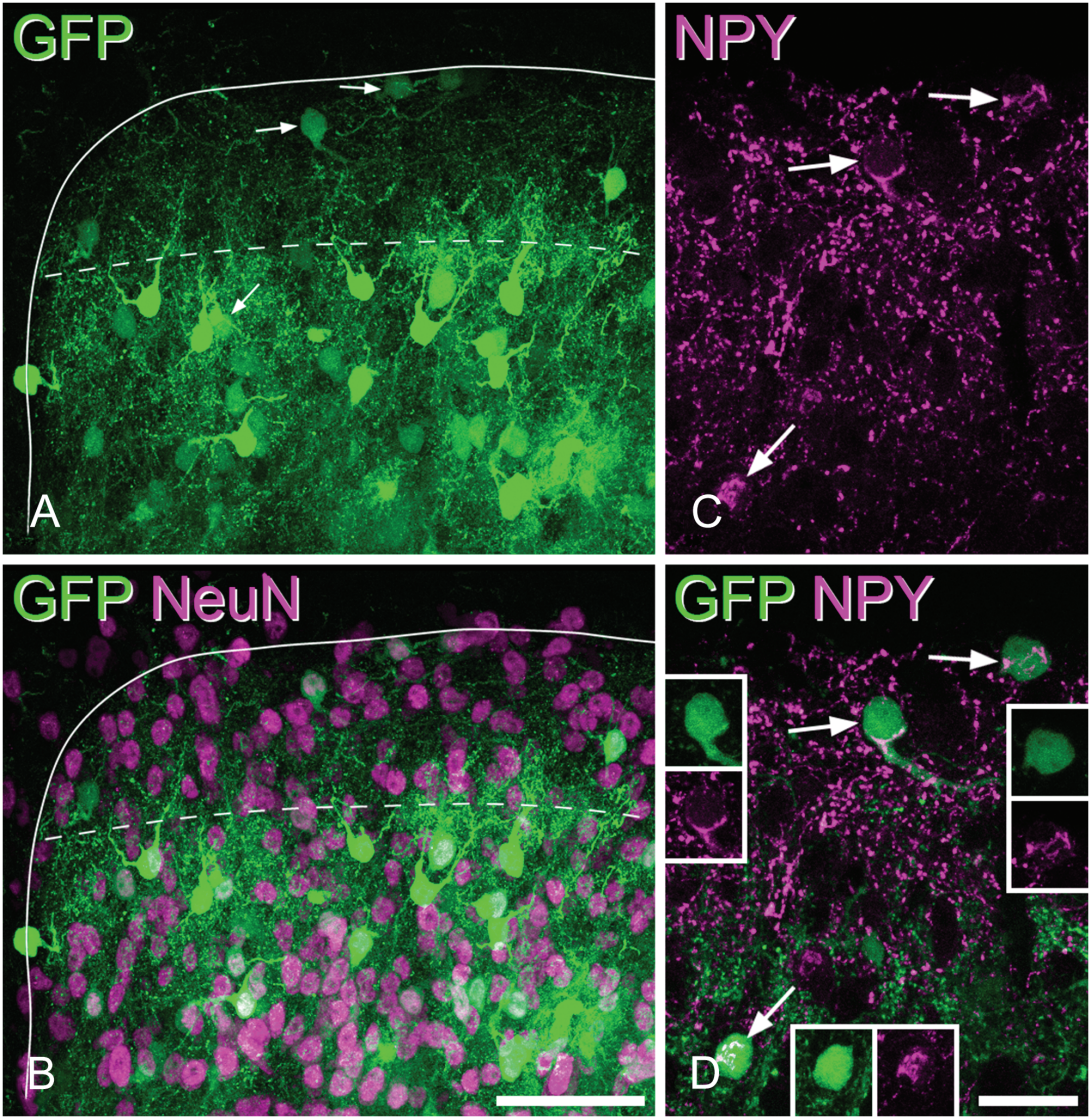
The distribution of green fluorescent protein (GFP) in the neuropeptide Y (NPY)-GFP mouse and its relation to NPY-immunoreactivity. (A and B) The medial part of the dorsal horn in a transverse section from a NPY-GFP mouse immunostained for NeuN (magenta). The solid line outlines the grey matter, and the dashed line shows the lamina II-III border. GFP^+^ cells (green) are present throughout this region but are more numerous in lamina III. (C and D) A higher magnification view of the same region scanned to reveal NPY (magenta) and GFP. NPY–immunoreactivity is present in many small structures, which correspond to axonal boutons, and also in the cell bodies of some neurons. Arrows point to 3 GFP^+^ cells that have NPY-immunoreactivity in their cell bodies, and the positions of these cells are shown with the arrows in (A). The insets show GFP (green) and NPY-immunoreactivity (magenta) separately in the cell bodies of each of these 3 cells. Note that the NPY staining is located in clumps within the perikaryal cytoplasm. All parts of the figure are maximum intensity projections of confocal z-stacks consisting of 46 (A, B) or 5 (C, D) optical sections at 1 μm z-spacing. Scale bars (A, B): 50 μm; (C, D): 20 μm.

We initially examined the relationship between GFP expression and NPY immunoreactivity in perfusion-fixed tissue from the NPY-GFP mouse. Although GFP^+^ cells were present throughout the dorsal horn, their density was relatively low in laminae I-II, while they were more numerous in lamina III (Fig 1A). Comparison with NeuN staining revealed that all GFP^+^ cells in laminae I-II and virtually all of those in lamina III were NeuN immunoreactive, confirming their neuronal identity (Fig 1B). However, in the deeper dorsal horn and ventral horn (laminae IV-IX), there were also cells that were weakly labelled with GFP and lacked NeuN, and these resembled glial cells. Immunostaining for glial fibrillary acidic protein confirmed that these were astrocytes (AJT, unpublished data).

Quantitative analysis (Table 2) revealed that the great majority (85%) of GFP^+^ cells throughout laminae I-III showed NPY immunoreactivity in their cell bodies (Figs 1C and D), and similar results were found when considering laminae I-II (78%) and lamina III (90%), separately. However, as expected from the relatively low density of GFP^+^ cells in the superficial dorsal horn, these accounted for only 33% of NPY-immunoreactive neurons in laminae I-II, whereas GFP was present in 82% of those in lamina III (Table 2).

**Table 2:**
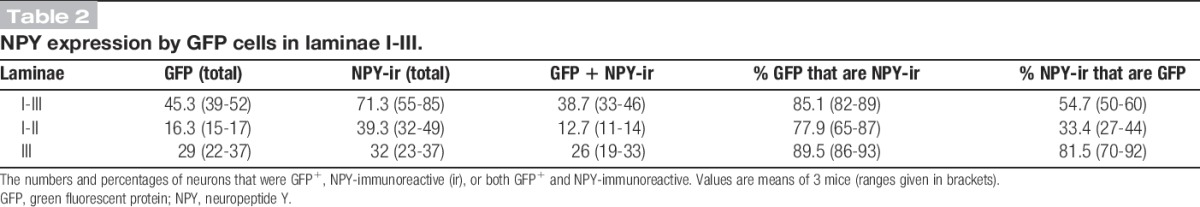
NPY expression by GFP cells in laminae I-III.

**Figure 2:**
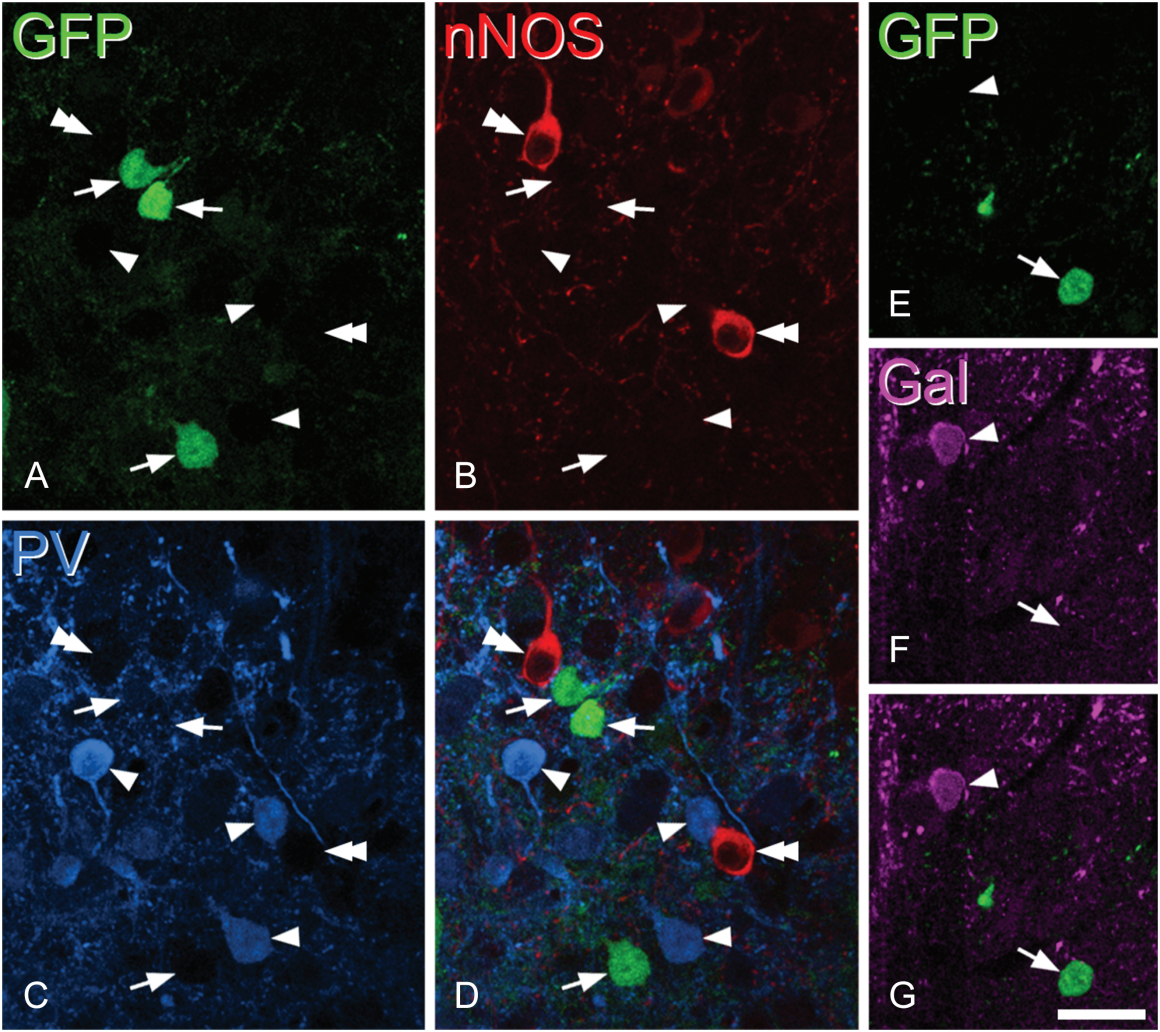
Lack of overlap between green fluorescent protein (GFP) and other markers for inhibitory interneuron populations. Confocal scans of a transverse section from a neuropeptide Y–GFP mouse that had been immunostained for neuronal nitric oxide synthase (nNOS) (red), parvalbumin (PV, blue), and galanin (Gal, magenta). A–D show 3 GFP^+^ cells (green, arrows) in lamina IIi-III that are not immunoreactive for either nNOS or PV but are surrounded by cells that are either PV-immunoreactive (arrowheads) or nNOS-immunoreactive (double arrowheads). (E–G) Part of lamina II from the same section scanned to reveal galanin and GFP. A GFP^+^ cell that lacks galanin (arrow) and a galanin-immunoreactive cell that lacks GFP (arrowhead) are shown. A–D consist of 3 optical sections at 1 μm z-spacing, while E–G are from a single optical section. Scale bar = 20 μm.

In the sections immunostained for galanin, nNOS, and parvalbumin (Fig 2), between 128 and 180 (mean, 156; n = 3 mice), GFP^+^ cells were identified in laminae I-III. None of these cells were immunoreactive for either nNOS or galanin, while a very small proportion (1.3%-2.3%; mean, 1.8%) were parvalbumin immunoreactive. Although we did not use a stereological method for this analysis, the general lack of colocalisation means that our results are unlikely to have been affected by any bias towards cells of different sizes.^16^

Penetration of all antibodies used in this part of the study was apparently complete because in each case, immunoreactive cell bodies were seen with approximately equal frequency throughout the depths of the sections.

### 3.2. Membrane properties of green fluorescent protein–positive cells recorded in the NPY-GFP mouse

The subthreshold *I*-*V* relationship, determined by giving brief voltage pulses (100 milliseconds; −70 to −50 mV; 2.5 mV increments), was used to calculate the resting membrane potential of each recorded cell. The average value across 96 cells was −51.1 ± 1.0 mV (SEM) with an input resistance of 1433.6 ± 79.8 MΩ. In current-clamp mode, cells were injected with incrementing depolarising current in the form of 1 second square pulses, and the first action potential evoked was analysed in detail. In 96 cells, the voltage threshold for evoking action potentials (defined as the point where rate of voltage rise exceeded 10 mV/milliseconds) was −33.6 ± 0.5 mV, the height of action potentials was 57.4 ± 1.7 mV, the base width was 3.6 ± 0.2 milliseconds, and the amplitude of after-hyperpolarisation was 30.3 ± 1.2 mV. Most recorded cells (89/96 cells, 93%) were able to generate action potentials repetitively, and these were defined as tonic (n = 81) or initial bursting (n = 8). A few (7/96, 7%) only produced 1 or 2 action potentials in response to suprathreshold current.

### 3.3. Primary afferent inputs to NPY-GFP cells

**Figure 3:**
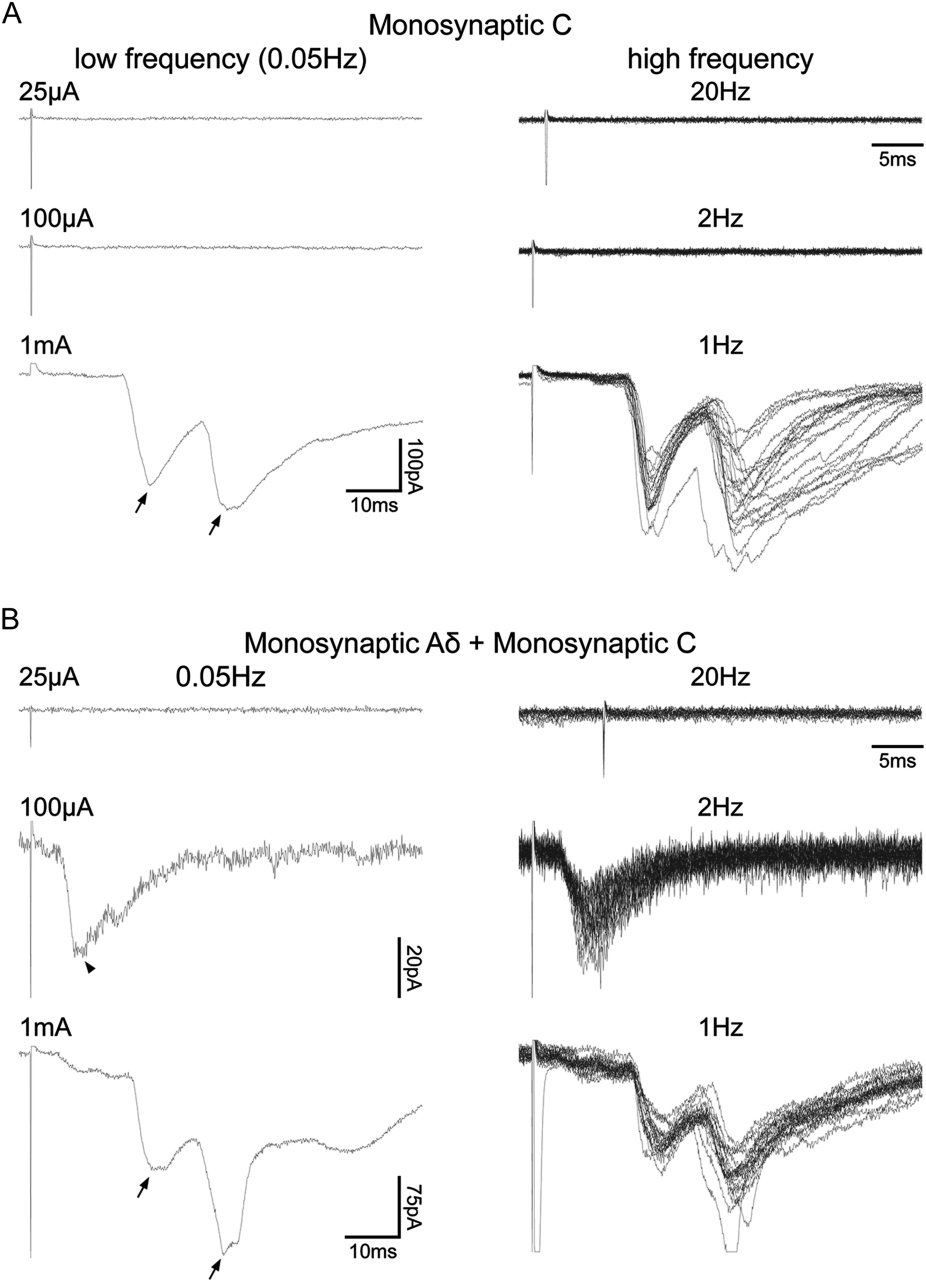
Primary afferent input to neuropeptide Y–green fluorescent protein (NPY-GFP) cells tested by dorsal root stimulation. (A and B) Characterisation of primary afferent input to NPY-GFP cells receiving monosynaptic C fibre only and monosynaptic Aδ with monosynaptic C-fibre input, respectively. Left panels show examples of evoked EPSCS (eEPSCs) resulting from low-frequency (0.05 Hz) dorsal root stimulation at Aβ-fibre (25 μA), Aδ-fibre (100 μA), and C-fibre (1 mA) intensities; each trace is an average of 3 recordings. Right panels show examples of eEPSCs resulting from high-frequency dorsal root stimulation (25 μA/20 Hz; 100 μA/2 Hz; 1 mA/1 Hz); each displays 20 superimposed traces. In both examples, 2 monosynaptic C-fibre inputs could be distinguished (each shown with an arrow). The monosynaptic Aδ input is indicated with an arrowhead.

Dorsal root stimulation was used to investigate primary afferent input to 39 of the NPY-GFP neurons, and this resulted in eEPSCs in 15 cells (38.5%). The remaining cells may have received primary afferent input from dorsal roots that were not stimulated or from axons that had been severed during the slice preparation. Of those cells with primary afferent input, 4 (26.7%) received polysynaptic C-fibre input only. The remaining 11 cells (73.3%) all received monosynaptic C-fibre input, with 8 of these cells receiving additional inputs that were monosynaptic Aδ (1 cell), polysynaptic C (2 cells), polysynaptic Aβ (2 cells), and polysynaptic Aβ with polysynaptic Aδ (3 cells). For 4 of the cells with monosynaptic C input, 2 distinct C-fibre eEPSCs could be distinguished (Fig 3). Stimulating dorsal roots at an increased intensity of 3 or 5 mA did not reveal any additional inputs in those cells that showed no monosynaptic response at 1 mA. The estimated conduction velocity of the monosynaptic C-fibre input was 0.14 ± 0.01 m/s, and the corresponding value for the single monosynaptic Aδ-fibre input was 0.49 m/s.

**Figure 4:**
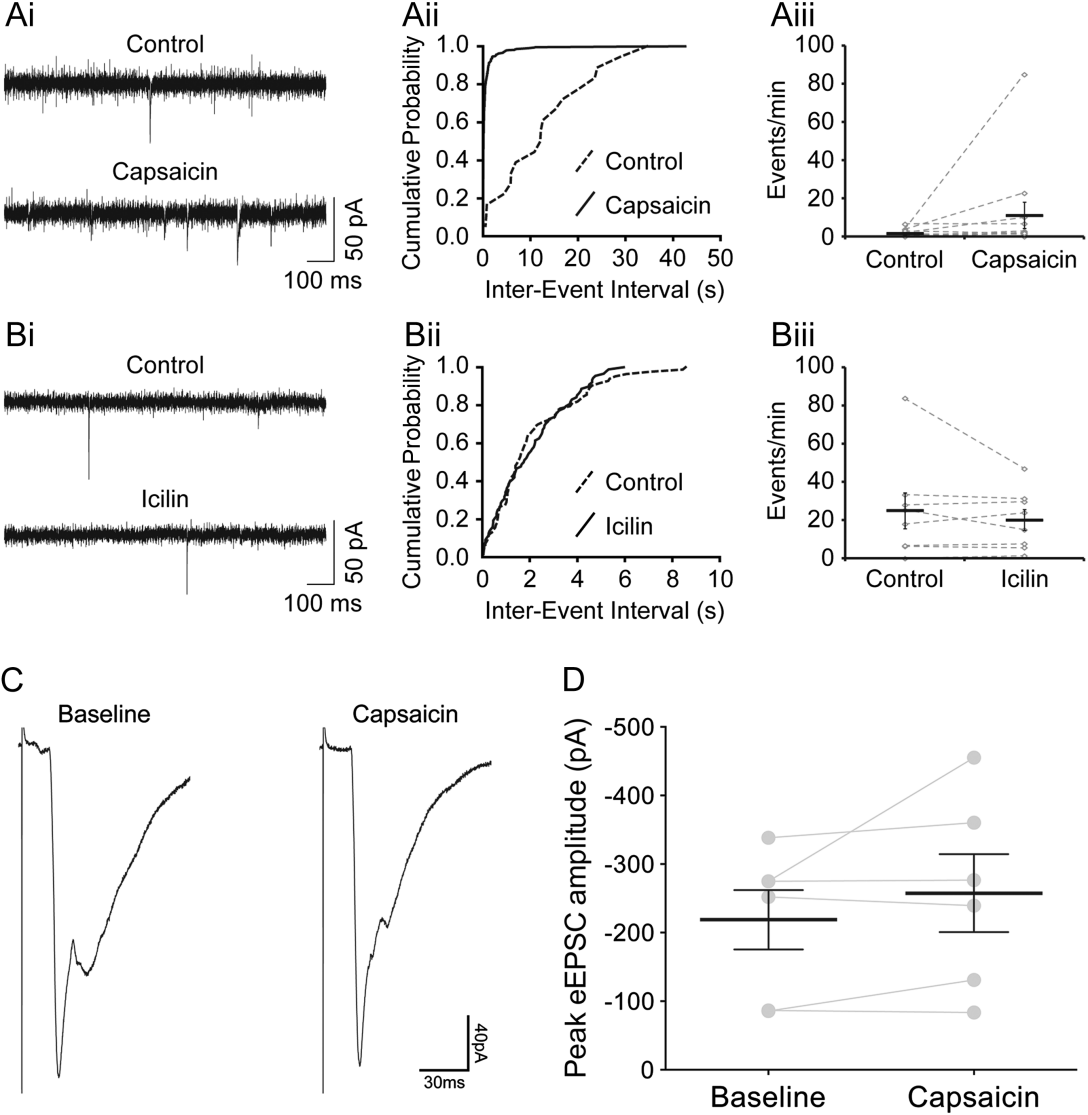
Effects of transient receptor potential channel agonists. (Ai) Voltage-clamp traces showing that the frequency of miniature EPSCs (mEPSCs) increased with bath application of capsaicin (2 μM). A significant increase in frequency was observed in 2 of 12 neuropeptide Y–green fluorescent protein (NPY-GFP) cells tested. (Aii) An example of a cumulative probability plot that shows a significant decrease in mEPSC interevent intervals (ie, an increase in mEPSC frequency). (Aiii) Changes in the rate of mEPSCs (events/min) with capsaicin application. The grey diamonds represent each cell tested, and the black horizontal bars show the average across the 12 cells. (Bi) Voltage-clamp traces in control conditions and in the presence of icilin (20 μM). Icilin did not cause any significant increase in mEPSC frequency in any of the 8 NPY-GFP cells tested. (Bii) An example of a cumulative probability plot showing no significant change in mEPSC interevent intervals with icilin application. (Biii) The rate of mEPSCs (events/min) in control conditions and in the presence of icilin. Grey diamonds represent the 8 cells tested, and the black horizontal bar shows the average values. (C and D) Capsaicin sensitivity of monosynaptic C-fibre input to NPY-GFP cells. (C) An example of monosynaptic C-fibre evoked EPSCs recorded in a NPY-GFP cell before (baseline) and during (capsaicin) application of capsaicin (2 μM). Baseline and capsaicin traces are an average of 9 recordings, corresponding to the 3 minutes before and the final 3 minutes of capsaicin application, respectively. (D) Of the monosynaptic C-fibre inputs tested for sensitivity to capsaicin, all 6 were found to be nonresponsive to capsaicin. Overall, capsaicin did not alter the peak amplitude of monosynaptic C-fibre input to NPY-GFP neurons (*n* = 6, *P* = 0.313, Wilcoxon matched-pairs signed-rank test). Data presented as mean ± SEM; grey points and lines indicate trajectories for individual cells.

Analysis of mEPSC frequency in response to bath application of transient receptor potential channel agonists suggested that most NPY-GFP cells do not receive monosynaptic inputs from either transient receptor potential vanilloid-1 (TRPV1)-expressing or transient receptor potential melastatin-8 (TRPM8)-expressing primary afferents. Application of a TRPV1 agonist, capsaicin (2 μM), led to an increase in mEPSC frequency in only 2 of 12 tested NPY-GFP cells (Fig 4A). Subsequent morphological analysis revealed that one of these cells had its soma in lamina IIo, while the other was in lamina III. The cell bodies of 5 of the cells that did not respond to capsaicin were identified: 2 of these were in lamina IIi and the other 3 were in lamina III. A TRPM8 agonist, icilin (20 μM), was tested on 8 cells, but none of these showed a significant increase in mEPSC frequency (Fig 4B). All neurons tested for icilin were recovered. Two of these had cell bodies located in lamina IIo, 4 in lamina Iii, and 2 were in lamina III.

The capsaicin sensitivity of the primary afferent input to NPY-GFP cells was assessed in a subset of those that received monosynaptic C-fibre input.^42,73^ Four cells, 2 of which had 2 separate monosynaptic C-fibre inputs, were tested. Application of capsaicin (2 μM) did not alter the peak eEPSC amplitude of any of the 6 monosynaptic C-fibre inputs (Figs 4C and D), which suggests that these resulted from activation of unmyelinated afferents that lacked TRPV1.^73^ Capsaicin did not evoke inward currents either in these cells or in those tested in the absence of dorsal root stimulation (see above).

### 3.4. Morphology of neurons recorded in the NPY-GFP mouse

**Figure 5:**
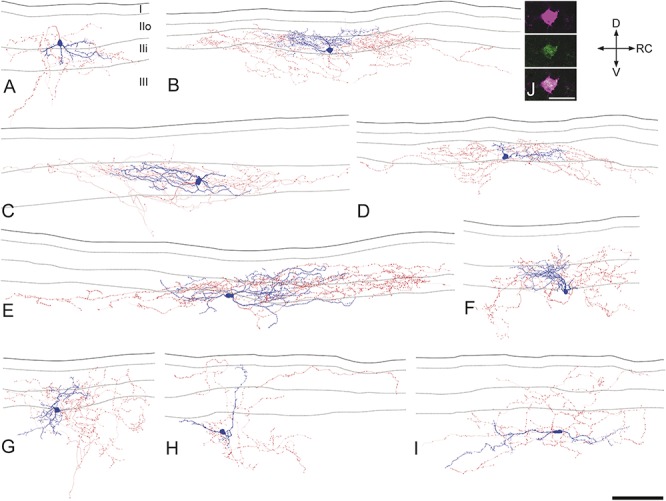
Morphology of recorded neuropeptide Y–green fluorescent protein (GFP) cells. (A–I) Examples of Neurolucida reconstructions for 9 of the recorded cells, 4 with cell bodies in lamina II (A–D) and 5 with cell bodies in lamina III (E–I). Cell bodies and dendrites are shown in blue and axons in red. In each drawing, the solid line indicates the grey-white border, while dashed lines represent the boundaries between laminae I, IIo, Iii, and III. Note the variability in dendritic and axonal morphology. The cells illustrated in (C) and (F) were both tested with dorsal root stimulation and correspond to (A) and (B) of Figure 4, respectively, and both have dendrites that arborise extensively in lamina II. (J) A confocal optical section through the soma of the cell illustrated in (B) shows Neurobiotin (magenta) and GFP (green). D, dorsal; RC, rostrocaudal; V, ventral. Scale bars: A–I, 100 μm; J, 20 μm.

Altogether, 65 of the recorded NPY-GFP cells were reconstructed with Neurolucida, and examples are shown in Figure 5. In all cases, GFP was detected in the cell body (Fig 5J). Twenty-three of these cells had their soma in lamina II (4 in lamina IIo and 19 in lamina IIi; Figs 5A–D), while for the remaining 42 cells, the soma was in lamina III (Figs 5E–I). The axonal arbors of all cells were well labelled, but in 7 cases (3 in lamina II and 4 in lamina III), the dendrites were very short and appeared to have been truncated. These cells were therefore excluded from the morphometric analysis of dendritic trees. The 58 cells with well-labelled dendritic trees were morphologically heterogeneous and did not fit into any of the classes that have been identified in previous studies.^15,75^ In particular, none of the recorded neurons were islet cells, which represent a well-defined class of inhibitory interneurons.^15,19,36,62,76^ The laminar locations of dendritic trees are shown in Table 3. For most cells (41/58), these occupied laminae II and III, but in 4 cases, they extended into lamina I. A few of the cells had dendritic trees restricted to a single lamina.

**Table 3:**
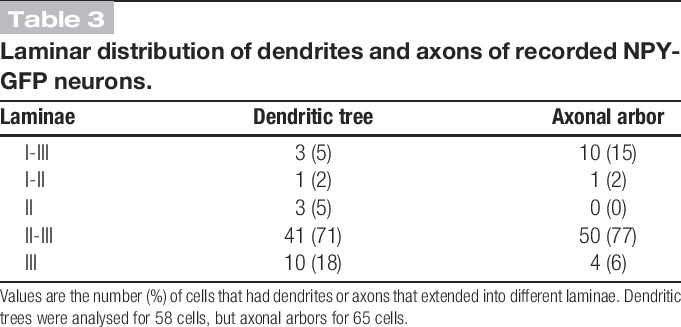
Laminar distribution of dendrites and axons of recorded NPY-GFP neurons.

The mean extents of the dendritic trees in rostrocaudal, dorsoventral, and mediolateral axes were 166, 90, and 50 μm, respectively (Table 4). This indicates that the cells are somewhat elongated in the rostrocaudal axis and occupy a relatively narrow mediolateral depth. When the corresponding values were compared between the cells with somata in lamina II and lamina III, the mean dorsoventral extent was found to differ significantly (68 and 101 μm, respectively; *P* < 0.01, unpaired t-test). We noticed that lamina III cells often had dorsally directed dendrites (eg, Figs 5F, H), and we therefore compared the extent of the dendritic tree that lay dorsal and ventral to the mid-point of the soma.^75^ The mean dorsal and ventral dendritic extents for the lamina III cells were 63 μm (± 36 μm) and 36 μm (± 29 μm), respectively, and these were significantly different (*P* < 0.01, unpaired t-test). A similar comparison for the lamina II cells showed that the dorsal and ventral dendritic extents did not differ significantly (dorsal, 38 ± 17 μm; ventral, 32 ± 24 μm; *P* = 0.46). This shows that the dendritic trees of the lamina III cells extend more dorsally than ventrally from the cell body and are consistent with the finding that 28 of the 38 lamina III cells had dendrites that extended into lamina II. In 9 cases, dorsal dendrites of lamina III cells entered lamina IIo. There was considerable variation in the complexity of dendritic branching, with some cells having highly branched dendritic trees (eg, Figs 5B, E) and others having dendrites that branched sparsely (eg, Figs 5A, H, I). The density of spines per 100 μm dendritic length varied considerably (0-13.6; median, 4), but there was no significant difference between cells in laminae II and III (*P* = 0.94, Mann–Whitney U-test). Morphological data were available for 9 of the 11 cells that received monosynaptic C-fibre input (see section 3.3 above), and 2 of these are illustrated in Figure 5 (cells C and F). Seven of these cells had cell bodies in lamina III, while the other 2 were in lamina IIi (eg, Fig 5C). In all cases, dendrites of these cells extended at least as far as the mid-part of lamina II (eg, Fig 5F). Among the cells that showed capsaicin-resistant monosynaptic C-fibre input, 3 were located in lamina III and 1 in lamina IIi. Two of these cells were located in the medial part of the dorsal horn, as judged by the presence of numerous dorsoventrally orientated myelin bundles.

**Table 4:**
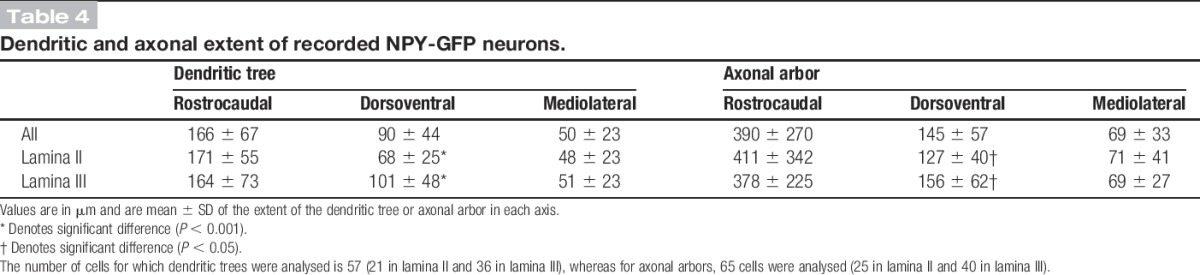
Dendritic and axonal extent of recorded NPY-GFP neurons.

**Figure 6:**
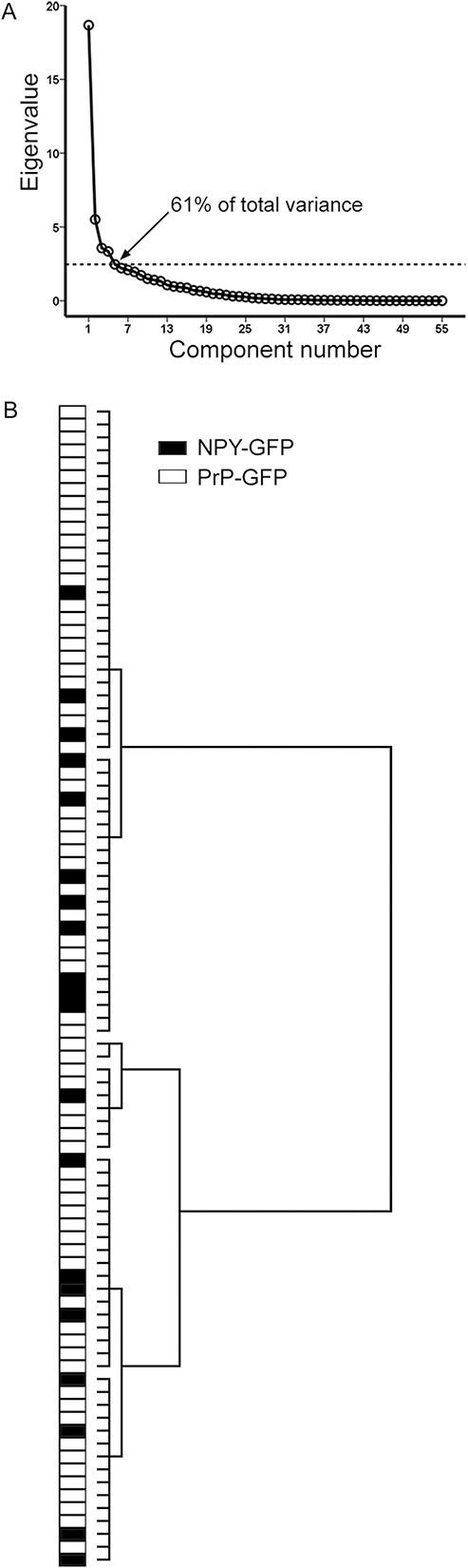
Cluster analysis of somatodendritic morphological parameters for neuropeptide Y–green fluorescent protein (NPY-GFP) neurons and PrP-GFP neurons in lamina II. (A) Scree plot of the eigenvalues derived from the principal component analysis of the 55 morphological parameters listed in Table 5. The first 5 factors accounted for 61% of the total variance. (B) Ward's method of unsupervised hierarchical clustering for the 20 NPY-GFP cells with well-labelled dendrites and cell bodies in lamina II, and 70 lamina II PrP-GFP cells reported in Ganley et al.^13^ Note that the NPY-GFP cells are interspersed among the PrP-GFP cells and are not found in separate clusters.

Inspection of the dendritic trees of the NPY-GFP cells in lamina II and those of the PrP-GFP cells described in our previous study^13^ revealed that both populations had highly variable morphology. To provide an objective comparison, we performed cluster analysis of these 2 populations, using the morphological parameters listed in Table 5. A scree test (Fig 6A)^6^ revealed that the decrease in eigenvalues reached a plateau at 5 principal components, and these were therefore used for cluster analysis. However, this analysis failed to separate the NPY-GFP cells into distinct clusters (Fig 6B). This suggests that these 2 groups of cells (NPY-GFP and PrP-GFP) do not belong to populations that are distinct from each other in terms of the somatodendritic morphology.

**Table 5:**
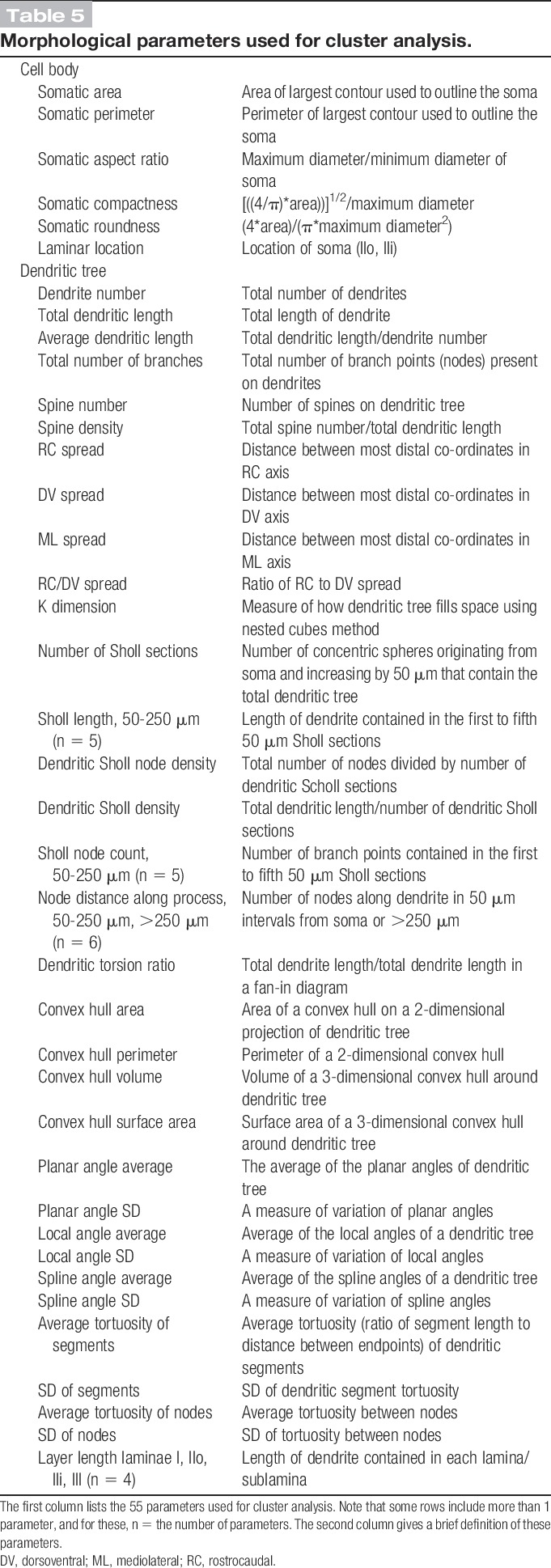
Morphological parameters used for cluster analysis.

Axons of most recorded cells (50/65) arborised in both laminae II and III, whereas in 4 cases, the axon was restricted to lamina III (Table 3). Eleven cells had axon entering lamina I, but 8 of these gave rise to <5 boutons in this lamina. The extents of axonal arbors in rostrocaudal, dorsoventral, and mediolateral axes are shown in Table 4. These were highly variable, with some being relatively extensive in the rostrocaudal axis (eg, Figs 5B–E), whereas others extended along both rostrocaudal and dorsoventral axes (eg, Figs 5A, F–I). The axonal arbors showed relatively little spread along the mediolateral axis (Table 4). The only significant difference between axons of cells in laminae II and III was that the latter had a greater dorsoventral extent (*P* < 0.05, unpaired t-test). The mean total length of the reconstructed axons for the 65 recorded cells was 4967 ± 2117 μm, and these possessed a mean of 560 ± 327 boutons, with a bouton density of 11.15 ± 4.07 per 100 μm length.

We have shown in both rat and mouse that ALT projection neurons in lamina III are densely innervated by NPY-immunoreactive boutons that are arranged in clusters that follow the dorsal dendrites of these cells.^5,48^ We have also provided evidence that these originate from a specific subset of NPY-expressing interneurons because these boutons were significantly larger and more strongly immunoreactive than other NPY-expressing boutons in this region.^47^ To identify the neurons giving rise to this input, we therefore used immunocytochemistry and tested whether axons of any of the recorded cells contributed to the bundles of NPY-immunoreactive axons that innervate the ALT cells.^47,48^ The cell bodies and dorsal dendrites of lamina III ALT cells are also densely innervated by peptidergic primary afferents, which can be revealed with antibodies against CGRP.^39^ The clusters of NPY axons that are associated with the ALT cells can therefore be recognised by their close association with bundles of CRGP-immunoreactive primary afferents.^5,48^

**Figure 7:**
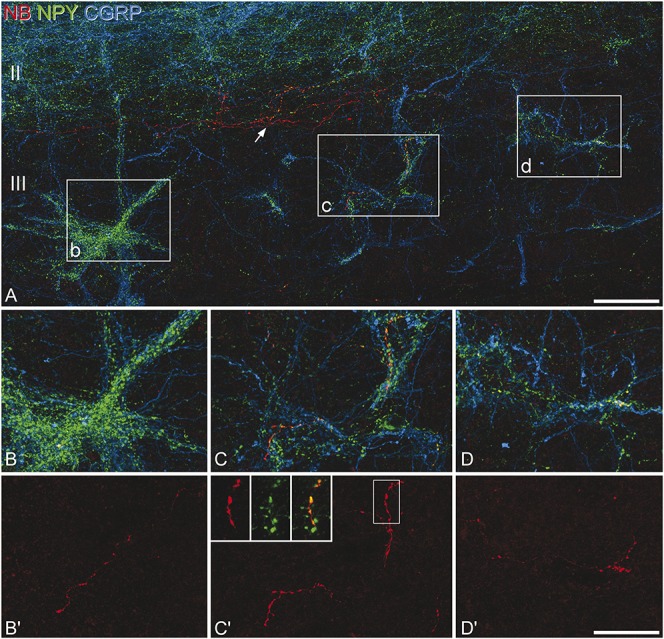
Innervation of neuropeptide Y (NPY) bundles by the axon of a recorded NPY–green fluorescent protein cell. (A) A projected confocal image stack (72 optical sections at 0.5 μm z-spacing) shows staining for NPY (green) and calcitonin gene-related peptide (CGRP, blue), together with Neurobiotin (NB, red) from a parasagittal section that contains part of the axon of a recorded cell. Axons containing NPY or CGRP form plexuses in lamina II (seen in the upper part of the image), and also distinct intermingled bundles in lamina III. Three of these bundles are located in the boxes, which correspond to the fields shown in (B), (C), and (D), and these are located approximately 150 to 200 μm apart along the rostrocaudal axis. (B–D) Higher magnification views of these 3 bundles. Note the presence of part of the axon of the recorded cell in each of these bundles, which can be seen more clearly when only NB is revealed (B'–D'). Many of the boutons belonging to this cell showed strong NPY-immunoreactivity, and this is illustrated in the inset in (C)' (projection of 12 optical sections at 0.5 μm z-spacing), which shows a magnified view of the area in the box. In addition to contributing to these bundles of NPY-immunoreactive axons, the axon of the recorded cell also gives rise to branches that are not associated with the bundles, indicated with the arrow in (A). Scale bars: (A) 50 μm; (B–D) 25 μm.

This analysis was performed on 38 of the recorded neurons. Sections that contained parts of the axonal arbor from the selected neurons were reacted with antibodies against NPY and CGRP. In these sections, intermingled bundles of NPY-immunoreactive and CGRP-immunoreactive axons were clearly visible in lamina III (Fig 7A), and these could often be followed dorsally into lamina II. We have shown that these bundles are associated with lamina III ALT cells retrogradely labelled from the lateral parabrachial area in the mouse.^5^ Examination of the reacted sections revealed that for the great majority of the cells that were tested (36 of 38), the axon of the recorded neuron did not contribute to any of these bundles of NPY-immunoreactive axons. However, in 2 cases, the axon was seen entering these bundles (Fig 7). In 1 case, 326 boutons were identified on the labelled axon, and 21 of these (6%) were located within a single bundle of NPY axons. For the other cell (illustrated in Fig 7), 104 of 340 boutons were located in bundles of NPY axons, and in this case, the axon was seen to contribute to 3 adjacent bundles, which were located ∼150 to 200 μm apart (Figs 7B–D). We also tested for the presence of NPY immunoreactivity in axonal boutons belonging to the recorded neurons, and this was found in 11 of the 38 cells. The lack of detectable NPY immunoreactivity in the other recorded cells is likely to reflect loss of the peptide, which can occur during whole-cell recording.^13^ The 2 cells that had axons contributing to the NPY bundles both showed strong NPY immunoreactivity in many of their boutons (Fig 7C').

### 3.5. pERK expression by NPY–immunoreactive cells

**Figure 8:**
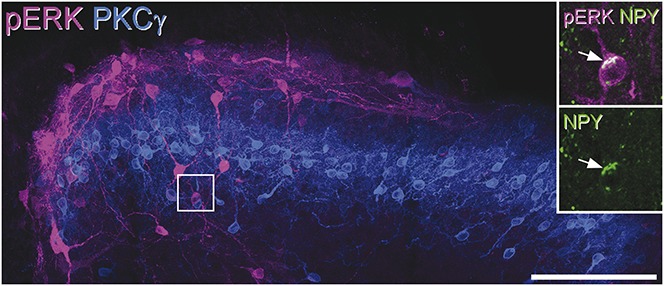
Phosphorylated extracellular signal-regulated kinase (pERK) expression after pinch stimulation. Transverse section through the dorsal horn from a mouse that received pinch stimuli to the ipsilateral hindpaw 5 minutes before perfusion, reacted to reveal pERK (magenta) and PKCγ (blue). The band of PKCγ profiles occupies lamina IIi. There are numerous pERK-immunoreactive cells in laminae I and IIo and scattered cells below the PKCγ band in lamina III. The boxed area is seen at higher magnification in the inset, which shows staining for neuropeptide Y (NPY) (green) in one of the pERK^+^ lamina III cells. The arrow indicates the NPY-immunoreactivity within the cell body. The main image is a projection of 22 optical sections at 1 μm z-spacing, while the inset is a projection of 6 sections at the same spacing. Scale bar = 100 μm.

Because NPY-GFP neurons in lamina III often had dendrites that extended into lamina II and some of these cells received monosynaptic input from C fibres, we tested whether NPY-expressing cells in lamina III responded to noxious stimulation. We used a pinch stimulus because we have found in the rat that this is particularly effective for generating pERK in lamina III neurons, including some that are NPY immunoreactive.^43^ As reported previously,^57^ 5 minutes after pinching of the skin on the hindpaw, pERK^+^ cells were seen in the medial part of the ipsilateral dorsal horn. The number and distribution of pERK^+^ cells varied considerably between sections, presumably reflecting the punctate nature of the pinch stimulus, and for this reason, we did not determine the proportion of NPY-immunoreactive cells that were pERK positive. Instead, we counted the total number of pERK^+^ and double-labelled (pERK^+^/NPY-immunoreactive) cells in lamina III in the 3 or 4 sections analysed from each mouse. Between 40 and 68 (mean, 57), pERK^+^ cells were identified in lamina III in the 3 mice, and between 4 and 9 of these cells were also NPY immunoreactive, corresponding to a mean of 11.7% (range, 10%-13.2%) of the pERK^+^ cells. An example is shown in Fig 8.

## 4. Discussion

The main findings of this study are (1) that NPY-GFP cells are morphologically diverse and that those in lamina II do not consistently fit into any of the morphological classes identified in this lamina,^15^ (2) that they include a specific subset with axons that contribute to the dense bundles of NPY axons that are associated with projection neurons in lamina III, and (3) that most of them were not innervated by TRPV1-expressing primary afferents.

### 4.1. Green fluorescent protein cells in the NPY-GFP mouse

As expected, the great majority of GFP^+^ cells in laminae I-III were NPY immunoreactive, and the lack of detectable NPY immunoreactivity in a small proportion of the cells probably results from a low level of peptide in cell bodies of some NPY-expressing neurons. This interpretation is consistent with the findings of van den Pol et al,^67^ who demonstrated that GFP in this mouse line was restricted to NPY-expressing neurons in several brain areas. Our finding that only a third of NPY-immunoreactive cells in laminae I-II expressed GFP was unexpected, and it is not known whether the GFP^+^ cells in laminae I-II are representative of all NPY-expressing interneurons in this region. However, the lack of colocalisation of GFP with galanin, nNOS, and parvalbumin clearly indicates that the NPY-GFP cells in laminae I-III are distinct from other population of inhibitory interneurons in this region.^61^

This is apparently the first morphological study of NPY-expressing cells in the dorsal horn and allows comparison with other neurochemically defined populations. Parvalbumin is expressed by inhibitory interneurons in laminae IIi and III, and many of these are islet cells,^1,24,33^ which indicates a clear morphological difference from the NPY-GFP neurons. Mesnage et al^37^ reported that cholinergic neurons in lamina III, which are a subset of the nNOS population,^59^ had a characteristic morphological appearance, with rostrocaudally elongated dendritic trees that often extended dorsally, thus resembling some of the lamina III NPY-GFP cells. We have recently described the morphological features of inhibitory interneurons that express GFP in the PrP-GFP mouse. These cells are mainly located in lamina II,^18^ and because 98% of them express nNOS and/or galanin,^27^ they are completely different from the NPY-GFP population. As with the NPY-GFP cells, the PrP-GFP cells were morphologically heterogeneous, but were never islet cells.^13^ Cluster analysis based on somatodendritic morphology failed to separate the 2 populations, and it is likely that neither contains cells with distinctive somatodendritic morphology. Despite this, there were differences between the populations in terms of their axonal projections and primary afferent inputs. Unlike the NPY-GFP cells, most PrP-GFP cells had axons that entered lamina I. In addition, most PrP-GFP cells showed an increase in mEPSC frequency after application of capsaicin and around half did so in response to icilin,^13,78^ which suggests that they are generally innervated by TRPV1-expressing nociceptors, and often by TRPM8^+^ cold-sensitive afferents. In contrast, increased mEPSC frequency was only seen on 2 of the 12 NPY-GFP cells tested with capsaicin and none of those tested with icilin. Therefore, although there are functional differences between the NPY-GFP and PrP-GFP populations, they cannot be distinguished based on somatodendritic morphology.

Taken together with the results of previous studies, our findings suggest that somatodendritic morphology cannot be used to define functional populations among those inhibitory interneurons that are not islet cells.

### 4.2. Innervation of lamina III ALT neurons

Anterolateral tract projection neurons with cell bodies in lamina III are densely innervated by axons that contain high levels of NPY and are believed to originate from a specific subset of NPY cells.^47^ We identified 2 cells that had axons with strong NPY immunoreactivity and contributed to dense bundles of NPY axons that were intermingled with CGRP bundles, an arrangement associated with the cell bodies and dendrites of lamina III ALT neurons.^5,48^ It is therefore highly likely that these cells innervated ALT neurons.

Cells of this type were rarely identified in our recordings, and there could be various explanations for this: (1) we may have failed to identify contributions to the NPY bundles from other recorded cells (although this seems unlikely because substantial parts of the axonal arbor were examined in most cases), (2) cells that innervate the ALT neurons may account for a small proportion of the NPY-expressing neurons, (3) they may be underrepresented among the cells that express GFP in this line, or (4) they may be relatively difficult to record from.

One of the 2 cells had an axon that contributed to 3 NPY bundles, which were located over 100 μm apart, and it is therefore likely that this cell innervated at least 3 different lamina III ALT neurons. For both cells, each axon accounted for only a small proportion of the boutons within the NPY bundles, which suggests that several NPY cells innervate each ALT neuron. We also found that in both cases, most axonal boutons belonging to the cell were located outside the NPY bundles, indicating that lamina III ALT neurons are not their only postsynaptic targets. PrP-GFP cells in lamina II give rise to axons that innervate projection neurons in lamina I, but we found that these cells also gave rise to numerous boutons in lamina II, which contains few dendrites of projection neurons.^13^ Taken together with the present findings, this suggests that although there are inhibitory interneurons that preferentially target specific types of projection cell, these projection cells represent only a minority of the synaptic output from the interneurons.

### 4.3. Functions of NPY–expressing interneurons

Because the NPY-expressing cells in laminae I-III are GABAergic,^3,22,50,71^ actions on their target neurons can be mediated through both GABAergic (synaptic) transmission and volume transmission involving NPY acting on Y1 and Y2 receptors in the dorsal horn.^4^ The actions of NPY are likely to be complex, as the Y1 receptor is present on a wide variety of spinal neurons, whereas both Y1 and Y2 receptors are expressed by primary afferents. A recent study reported that NPY depletion in adult mice did not alter acute pain thresholds but could prolong or reinstate hyperalgesia and allodynia in neuropathic and inflammatory models.^58^ This suggests that NPY may have little effect on sensory processing under normal conditions, but that tonic release is antinociceptive in pathological pain states. However, interpreting the mechanisms of action of NPY in chronic pain is complicated because it is upregulated in primary afferents after peripheral nerve injury,^68^ and in dorsal horn neurons in inflammatory pain states.^30^ It is therefore not known how much of its antinociceptive action results from peptide released by the NPY-expressing inhibitory interneurons.

Although we seldom found NPY-GFP cells with monosynaptic input from TRPV1-positive afferents, we had previously reported that 40% of NPY-immunoreactive neurons in laminae I-II of the rat showed pERK in response to capsaicin.^46^ In adult mice, TRPV1 is expressed by peptidergic C nociceptors, but not by those that lack neuropeptides and express the Mas-related protein Mrgd,^79^ whereas in rat, TRPV1 is expressed by both populations.^7^ The apparent species difference in responsiveness to capsaicin may therefore reflect innervation of NPY cells by nonpeptidergic C nociceptors, which would express TRPV1 in rat, but not mouse. We found that most NPY cells in lamina III had dorsally directed dendrites that entered the superficial dorsal horn (where C fibres terminate) and that some of these received monosynaptic C-fibre input. Among the potential sources for capsaicin-insensitive (TRPV1−) C-fibre input are TRPM8^+^ cold-sensitive afferents,^9^ nonpeptidergic (C-Mrgd^+^) nociceptors,^79^ and C–low-threshold mechanoreceptors.^55^ Our finding that NPY-GFP cells did not respond to icilin indicates that they are unlikely to be innervated by cold-sensitive afferents. Although we could not distinguish between the other 2 possibilities, some of the cells with capsaicin-insensitive monosynaptic C-fibre input were located in the medial part of the L4-5 segments, an area that is innervated by glabrous skin, which lacks C–low-threshold mechanoreceptors.^34^ It is therefore likely that some lamina III NPY cells are innervated by C-Mrgd afferents, which are believed to be important for mechanical nociception.^8^ This is consistent with the activation of lamina III NPY cells by mechanical noxious stimuli, as revealed with pERK. Taken together with our previous findings,^46^ these results suggest that many of the NPY neurons in laminae I-II, and also some of those in lamina III, respond to noxious stimuli and that these responses are transmitted at least in part by C-Mrgd afferents. The targets of the NPY cells include nociceptive-activated projection neurons,^43^ and also interneurons in the superficial laminae. They would therefore be well-placed to attenuate pain and limit its spread from the site of injury,^51^ by inhibiting excitatory interneurons and projection cells in nociceptive pathways, both through their GABAergic synapses and through NPY receptors. Because many of the NPY-expressing inhibitory interneurons are likely to respond to noxious stimuli, they may be less important for the separation of sensory modalities^51^ that prevents tactile stimuli from being perceived as painful under normal conditions.^72^

## Conflicts of interest

The authors report no conflicts of interest.
